# Customized Massive Parallel Sequencing Panel for Diagnosis of Pulmonary Arterial Hypertension

**DOI:** 10.3390/genes11101158

**Published:** 2020-09-30

**Authors:** Jair Antonio Tenorio Castaño, Ignacio Hernández-Gonzalez, Natalia Gallego, Carmen Pérez-Olivares, Nuria Ochoa Parra, Pedro Arias, Elena Granda, Gonzalo Gómez Acebo, Mauro Lago-Docampo, Julian Palomino-Doza, Manuel López Meseguer, María Jesús del Cerro, Spanish PAH Consortium, Diana Valverde, Pablo Lapunzina, Pilar Escribano-Subías

**Affiliations:** 1 Institute of Medical and Molecular Genetics (INGEMM)-IdiPAZ, Hospital Universitario La Paz-UAM Paseo de La Castellana, 261, 28046 Madrid, Spain; jairantonio.tenorio@gmail.com (J.A.T.C.); nataliagallegozazo@gmail.com (N.G.); palajara@gmail.com (P.A.); elena.granda@estudiante.uam.es (E.G.); gonzalo.gomezacebo@gmail.com (G.G.A.); spanishpahconsortium@gmail.com (S.P.C.); 2 CIBERER, Centro de Investigación Biomédica en Red de Enfermedades Raras, ISCIII, Melchor Fernández Almagro, 3, 28029 Madrid, Spain; 3 ITHACA, European Reference Network on Rare Congenital Malformations and Rare Intellectual Disability, 1000 Brussels, Belgium; 4 Department of Cardiology, Hospital Universitario Río Hortega, 47012 Valladolid, Spain; hgnacho@hotmail.com; 5 Unidad Multidisciplinar de Hipertensión Pulmonar, Servicio de Cardiología, Hospital Universitario 12 de Octubre, 28034 Madrid, Spain; carmenperezolivaresd@gmail.com (C.P.-O.); nuriaochoaparra@hotmail.com (N.O.P.); pilar.escribano.subias@gmail.com (P.E.-S.); 6 CINBIO, Universidade de Vigo, 36310 Vigo, Spain; maurolagodocampo@gmail.com (M.L.-D.); dianaval@uvigo.es (D.V.); 7 Instituto de Investigación Sanitaria Galicia Sur, Hospital Álvaro Cunqueiro, 36213 Vigo, Spain; 8 Centro de Investigaciones Biomédicas (CINBIO), 36310 Vigo, Spain; 9 J. CIBERCV, Centro de Investigación Biomédica en Red de Enfermedades Cardiovasculares, ISCIII, 28029 Madrid, Spain; julian.palomino@salud.madrid.org; 10 Unidad de Miocardiopatías Familiares, Servicio de Cardiología, Hospital Universitario 12 de Octubre, 28041 Madrid, Spain; 11 Servicio de Neumología, Hospital Universitario Vall d’Hebron, 08035 Barcelona, Spain; manuelopez@vhebron.net; 12 Paediatric cardiology and Grown up Congenital Heart Disease Unit, Hospital Ramón y Cajal, 28034 Madrid, Spain; majecerro@yahoo.es

**Keywords:** pulmonary arterial hypertension, massive parallel sequencing, NGS, digenic inheritance, and genetics

## Abstract

Pulmonary arterial hypertension is a very infrequent disease, with a variable etiology and clinical expressivity, making sometimes the clinical diagnosis a challenge. Current classification based on clinical features does not reflect the underlying molecular profiling of these groups. The advance in massive parallel sequencing in PAH has allowed for the describing of several new causative and susceptibility genes related to PAH, improving overall patient diagnosis. In order to address the molecular diagnosis of patients with PAH we designed, validated, and routinely applied a custom panel including 21 genes. Three hundred patients from the National Spanish PAH Registry (REHAP) were included in the analysis. A custom script was developed to annotate and filter the variants. Variant classification was performed according to the ACMG guidelines. Pathogenic and likely pathogenic variants have been found in 15% of the patients with 12% of variants of unknown significance (VUS). We have found variants in patients with connective tissue disease (CTD) and congenital heart disease (CHD). In addition, in a small proportion of patients (1.75%), we observed a possible digenic mode of inheritance. These results stand out the importance of the genetic testing of patients with associated forms of PAH (i.e., CHD and CTD) additionally to the classical IPAH and HPAH forms. Molecular confirmation of the clinical presumptive diagnosis is required in cases with a high clinical overlapping to carry out proper management and follow up of the individuals with the disease.

## 1. Introduction

Pulmonary arterial hypertension (PAH) is an uncommon disease, characterized by the increase in blood pressure in the lung arteries, which causes right ventricle failure and potentially can lead to death if not treated. According to the Spanish PAH Registry (REHAP), the prevalence of the disease in individuals older than 14 years is approximately 15 to 50 cases per million of the population [1]. 

Pulmonary hypertension (PH) classification has been recently proposed and encompasses five groups [2]. Classically, PAH included in group one diagnosis was based on hemodynamic parameters assessed by right heart catheterization: mean pulmonary arterial pressure (mPAP) ≥ 25 mmHg at rest, pulmonary artery wedge pressure (PAWP) ≤ 15 mmHg, and pulmonary vascular resistance (PVR) > 3 UW, in the absence of other causes of precapillary PH such as PH due to lung diseases, chronic thromboembolic disease or other rare diseases [3].

Idiopathic (IPAH) and heritable PAH (HPAH), included within group one, have been classically associated with *BMPR2* mutations [4], with a penetrance of 20%, which means that approximately 80% of individuals with *BMPR2* will not develop clinically detectable PAH [5]. Mutations in this gene only explain 20% of idiopathic cases and about 60% of heritable PAH individuals [5,6], suggesting that additional genes are responsible for PAH. Thus, several genes were reported in the past few years associated with PAH. 

Genes described in PAH are associated with a variety of molecular pathways [6,7,8,9,10,11,12]. Currently, 12 genes have been clearly related with PAH with a higher level of evidence: *BMPR2*, *EIF2AK4*, *TBX4*, *ATP13A3*, *GDF2*, *SOX17*, *AQP1*, *ACVRL1*, *SMAD9*, *ENG*, *KCNK3*, and *CAV1*, and five with a lower level of evidence: *SMAD4*, *SMAD1*, *KLF2*, *BMPR1B*, and *KCNA5* [13]. Also, there is increasing evidence for the involvement of other genes in PAH with less frequency and in different pathways. *KLK1* and *GGCX* have been recently related to IPAH [14].

Pulmonary venooclusive disease (PVOD) is the most lethal subtype of PAH, with different clinical, histological, and genetic features. It is characterized by very low diffusion capacity (DLCO), resting hypoxemia, severe desaturation on exercise, and a characteristic radiological pattern. Heritable forms are caused by bi-allelic mutations in *EIF2AK4*, a member of the kinases that phosphorylate the alpha subunit of eukaryotic translation initiation factor-2. We showed that consanguineous families with PVOD from Gipsy origin have a founder effect mutation in *EIF2AK4*, which causes a missense variant and haploinsufficiency of the gene [15]. Nowadays PVOD and pulmonary capillary disease are considered similar entities with overlapping clinical features, with the same underlying genetic mechanism [16]. Differential diagnosis between PAH and PVOD remains a clinical challenge and misdiagnosis is a common problem with important clinical implications. 

More recently, a “second hit” hypothesis came up due to the identification of two pathogenic variants in two genes related to PAH [17]. Eichstaedt et al. identified a family with two variants, one in *EIF2AK4* and one in *BMPR2*, in a family with HPAH [18]. Additionally, the group of Yuxin Fan described a severe early-onset PAH individual that had two variants probably contributing to the disease, one in *BMPR2* and one in *KCNA5* [19]. This was also reported in two families from Lebanon in a combination of variants in *BMPR2-GDF2* and *BMPR2-TBX4*, respectively [20]. Taking this into account and based on the low penetrance of *BMPR2*-associated PAH, the co-occurrence of more than one variant associated with the disease might explain why some individuals with only *BMPR2* mutations do not develop PAH. This can explain in part why the presence of *BMPR2* pathogenic variants alone is not always enough to develop the disease.

Thus, the main objective of this study was to study a cohort of three hundred patients with idiopathic, heritable, PVOD and APAH from the Spanish registry of patients with PAH (REHAP), through a custom, in-house NGS panel of 21 genes. Secondly, phenotype–genotype correlation and survival rate were also assessed.

## 2. Material and Methods

### 2.1. Cohort Description

All patients from group one and idiopathic, heritable, and PVOD and patients with associated PAH to congenital heart disease and connective tissue disease included in the national Spanish Registry of Pulmonary Arterial Hypertension (REHAP) were eligible for this study (Supplementary Figure S1). REHAP is an observational national registry with the aim to serve as a database for research purposes. It includes patients with any form of PAH [21,22]. Clinical parameters for PAH diagnosis were obtained from the 2015 ERS/ESC guidelines [3].

In Spain, genetic testing of patients included in the REHAP has been offered since 2011, with a focus on IPAH, HPAH, and PVOD at the beginning but later expanded to associated forms as well. This study followed the ethical principles of the European board of medical genetics. All patients signed a consent form and genetic counseling pre and post-test was offered.

The study was approved by the ethical committee for scientific research of each participant center and also by the ethical committee of the La Paz University Hospital (CEIC-HULP PI-1210).

Before genetic testing, family history information was collected, and DNA samples from the probands were extracted. Segregation analysis in patients carrying a variant of unknown significance was performed when possible. In cases of unaffected carriers, a complete diagnostic and specific follow up was performed to rule out PAH. 

### 2.2. Statistical Analysis

Categorical variables are presented as absolute frequency and proportions, and continuous variables as the mean ± standard deviation or median (interquartile range). In order to validate the normality of the statistical distribution, the Kolmogorov–Smirnov test was applied, and to compare clinical features and continuous variables among patients, t-student was calculated. For variables that did not follow a normal distribution, the Wilcoxon signed-rank test was used. The rate of percentages was compared by Chi-square test, Fisher exact test, or Wilcoxon test, as appropriate.

Survival analysis was performed using the Kaplan-Meier analysis, matching the date of diagnosis with the date of the first diagnostic right heart catheterization (RHC). All-cause mortality or bilateral lung transplantation were defined as the endpoint and the log-rank test was used for comparison between groups. Multivariate Cox regression models identified significant predictors of mortality. A comparison of the survival rate among different groups was also performed (Supplementary Figure S2).

A two-sided *p*-value of less than 0.05 was considered statistically significant. Statistical analyses were done using Stata (v12.1 for Mac; StataCorp, College Station, Brazos County, TX, USA). 

### 2.3. Genetic Analysis

A customized panel of 21 genes (HAP v1.2) including *ABCC8*, *ACVRL1*, *BMPR1B*, *BMPR2*, *CAV1*, *CBLN2*, *CPS1*, *EIF2AK4*, *ENG*, *GDF2*, *KCNA5*, *KCNK3*, *MMACHC*, *NOTCH3*, *SARS2*, *SMAD1*, *SMAD4*, *SMAD5*, *SMAD9*, *TBX4*, and *TOPBP1*. This custom panel was designed with NimbleDesign (Roche, Indianapolis, IN, USA). Fragmentation and library preparation were performed with SeqCap EZ Choice Enrichment Kit (Roche, Indianapolis, IN, USA). Sequencing was performed with the Illumina MiSeq platform (Illumina, San Diego, CA, USA) following the manufacturer’s instructions. Genes were chosen because of their probed association with PAH or based on previous research data. An in-house pipeline for bioinformatic analysis was developed to perform quality control and variant annotation. 

Our Institute (INGEMM) has developed a suite of quality control (QC) scripts that simplify data quality assessment. QC included an assessment of total reads, library complexity, capture efficiency, coverage distribution (95% at ≥20×), capture uniformity, raw error rates, Ti/Tv ratio in coding regions (typically 3.2 for known sites and 2.9 for novel sites), and distribution of known and novel variants relative to dbSNP and zygosity.

Annotation: an automated pipeline for the annotation of variants derived from targeted sequences in the capture genes was developed. INGEMM script application returned several variant annotations including dbSNPrs identification, gene names with accession numbers, predicted functional effect, protein positions and, for amino-acid changes (dbNSFP, CADD), conservation scores and several population frequency databases, and known clinical associations along with a vast array of annotations for non-coding sequences derived from ENCODE. Databases for pathogenic variants such as ClinVar, Human Gene Mutation Database (HGMD), Mastermind, LOVD, Alamut, and Varsome have also been reviewed.

**Figure 1:**
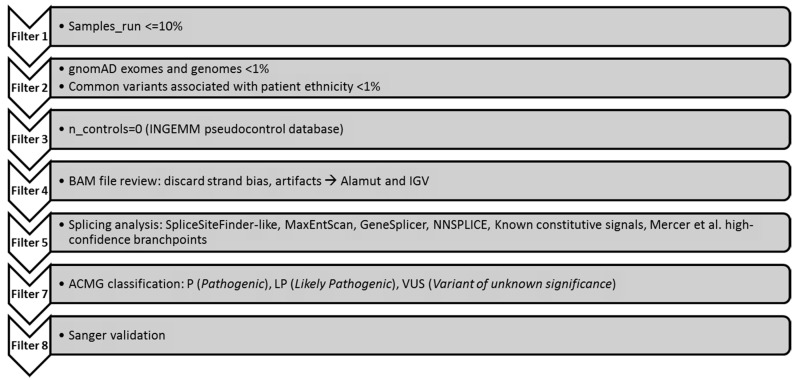
Filtering pipeline for NGS panel HAP v1.2. A set of filters applied for variant prioritization. Only variants does not fulfil the criteria for variant quality were validated with Sanger sequencing [25].

After that, the variant prioritization was performed according to the filtering strategy described in Figure 1. Finally, variants were classified according to the ACMG guidelines [23]. A custom script developed in-house called “LACONv” has also been developed by INGEMM in order to analyze large genomic DNA gain and losses or copy number variation (CNVs) which can be found in the following repository (https://github.com/kibanez/LACONv) [24]. 

## 3. Results

After the quality control analysis, 33 out of the 300 sequenced samples were discarded and 267 were subsequently filtered. We have identified 86 variants in 81 patients (32.2%). Out of them, 34 (39.5%) have been classified as pathogenic variants, 14 (16.3%) as likely pathogenic, and 38 (44.2%) as variants of unknown significance (VUS). In 186 samples non pathogenic, likely pathogenic, or VUS were detected in the analyzed genes. *BMPR2* was the predominantly mutated gene with 25 pathogenic or likely pathogenic variants (29%, 25/86) followed by *EIF2AK4*, *TBX4*, and *ACVRL1*. 

**Figure 2:**
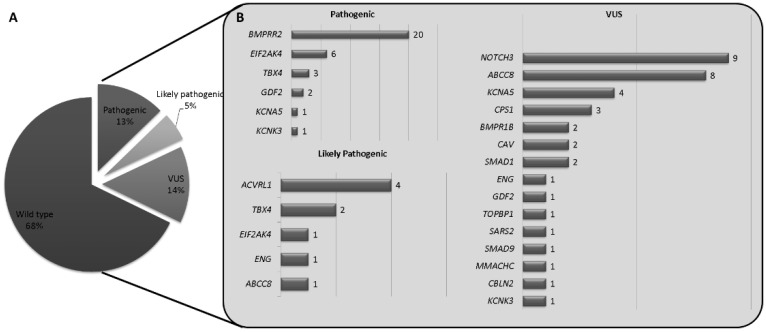
Frequency of gene mutation detected after the analysis of 21 genes. (**A**) A total of 14% of pathogenic and likely pathogenic variants were identified, with a 15% of variants of unknown significance (VUS) and in approximately 65% of individuals no candidate gene variants were identified. After quality check, 6% of the analyzed samples were discarded due to low quality. (**B**) The gene with the highest pathogenic variants detected was BMPR2, followed by EIF2AK4, because many patients with PVOD were also included in the analysis.

Regarding the VUS variants detected, *NOTCH3* and *ABCC8* had the highest frequency of these variants (Figure 2). Based on the etiology, 41 variants were detected in 38 IPAH individuals, 15 in 15 individuals with HPAH, and 10 in 8 individuals with PVOD. In associated -PAH, we have found 15 variants (Supplementary Tables S1 and S2). Finally, in all three patients with suspected heritable hemorrhagic telangiectasia (HHT), an *AVCRL1* pathogenic variant was detected, confirming the clinical suspicion. 

**Figure 3:**
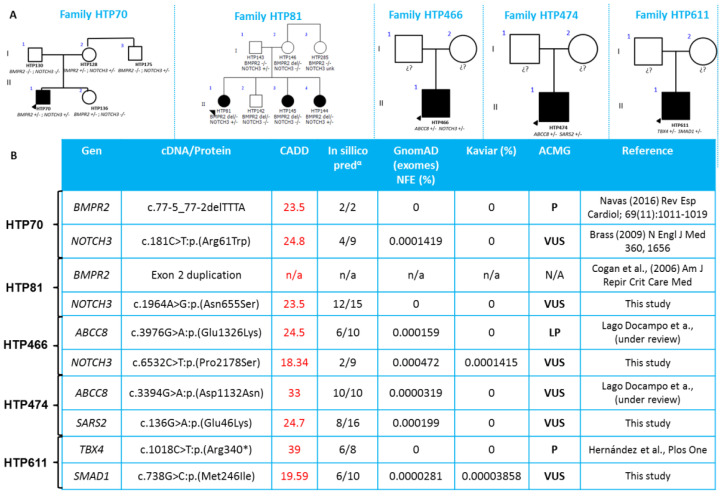
Pedigree and in silico analysis of the families with more than one variant detected. (**A**) Genealogy of the families in which segregation analysis was performed (when available). (**B**) Table of in sillico analysis of the candidate variants, including the ACMG classification as well as the reference in which the variant was described. n/a: not applicable, P: pathogenic, VUS: Variant of unknown significance. α: In silico analysis was performed by dbNSFP counting the ones that predict a deleterious effect over the total available.

Strikingly, we have found five unrelated families in whom there was more than one candidate causative variant (Table 1, Figure 3). In two families, one of the variants was found in *BMPR2* and the co-occurrence was with *NOTCH3* (x2). In the other three families, the variants were located in *ABCC8-NOTCH3*, *ABCC8-SARS2*, and *TBX4-SMAD1*. Three of these families were classified as IPAH and the other two as PAH-CHD. 

In all consanguineous individuals with a clinical diagnosis of PVOD, we found the known Spanish founder missense pathogenic variant in *EIF2AK4*:NM_001013703.3:c.3344C>T(p.Pro1115Leu) in a homozygous state [15]. Additionally, in two siblings from a non-consanguineous couple, we found compound heterozygous pathogenic variants in *EIF2AK4* ((*EIF2AK4*:NM_001013703.3(EIF2AK4_v001):c.3766C>T:p.(Arg1256*) and *EIF2AK4*:NM_001013703.3(EIF2AK4_v001):c.4392dup:p.(Lys1465*)). Segregation analysis demonstrated that parents were carriers of each variant. One of the siblings is a Caucasian male with a clinical presumptive diagnosis of PVOD at 30 years of age. He underwent bilateral lung transplantation 3.5 years after diagnosis. His sister was also diagnosed with PVOD at 38 years of age leading to lung transplantation one year after diagnosis. 

We have found five patients from five unrelated families carrying a pathogenic or likely pathogenic variant in *TBX4* [24]. All patients had a variable clinical expressivity with a moderate to severe reduction in diffusion capacity (DLCO).

Finally, in *CBLN2*, we found one candidate variant in a patient with IPAH classified as VUS (*CBLN2*: NM_182511.3:c.263C>T:p.(Ser88Phe)).

## 4. Discussion

PAH is a multifactorial disease, in which many different phenotypes and etiologies can explain the prognosis and severity of the disease. During the last few years, mainly due to the advances in genomics technologies, many genes and genomic associations were described in PAH. Therefore, nowadays the diagnostic approach for PAH should include genetic analysis because it is crucial for follow up, genetic counseling, to grade the severity, or even for future personalized therapies [26,27]. Since 2014, we have been evaluating patients not only with IPAH, HPAH, and PVOD but also secondary forms from which there is not so much evidence for the relation with genetic mutations.

Thus, the HAP-panel v1.2 included 21 genes based on the literature, previous experience, and well-established genes in PAH, which influence the pathophysiology of the disease. We included patients with PAH, no matter the type of PAH (idiopathic, heritable, or associated forms). We found that 30.34% (81/267) of individuals with pathogenic, likely pathogenic, and/or VUS variants (Figure 2). A total of 53.41% (47/88) of these variants were classified as pathogenic or likely pathogenic, and the remaining 31.82% (38/88) VUS, meaning that we have a relatively high number of variants without clear effect. The majority of the VUS have been found in *NOTCH3*, a gene from which mutations have been recently associated with IPAH [9,28,29]. It has been suggested that *NOTCH3* mutations are involved in proliferation and cell viability and impairs the NOTCH3-HES5 signaling pathway, a crucial pathway in the development of PAH [30].

Regarding *NOTCH3*, it has been suggested that the majority of the mutations in *NOTCH3* cause a missense change in the protein. Out of the 38 VUS variants reported herein, 23.7% (9/38) have been found in *NOTCH3*, which means that functional studies and segregation analysis in available cases are strongly recommended to confirm or discard the possible effect for these variants in the protein function and pathways in which *NOTCH3* is involved. 

This means that, if all the *NOTCH3* variants were classified as pathogenic or likely pathogenic, the frequency of mutations in this gene in PAH would be much more common than initially reported [9]. Strikingly, among the five unrelated families with a suggested digenic inheritance, two have had pathogenic variants in *BMPR2* and *NOTCH3*, which also segregates with the disease (Table 1). One of the index patients was classified as IPAH and clinical features of the proband showed one adult diagnosed at 39 years, with severe hemodynamic parameters (very low cardiac output). Oral dual therapy (riociguat and ambrisentan) was initiated with clinical and hemodynamic improvement. Eight years after diagnosis, he presents a low risk of death under dual oral therapy. It is important to remark that, as far as we know, there is no previous evidence of segregation of variants in *BMPR2* and *NOTCH3*, and this fact, might explain in part, why penetrance of *BMPR2* in idiopathic cases of PAH is only about 20% (14% in males and 42% in females) [6,13].

Furthermore, three additional families have been found to have two variants in two genes (Table 1, Figure 3); adding evidence that digenic inheritance may be a mechanism associated with PAH as a previous report suggested [18]. Two out of these three families were diagnosed with APAH-CDH and one with IPAH. 

Two siblings from an unrelated couple were found to have a compound heterozygous mutation in *EIF2AK4*, each one inherited from healthy parents. Clinical features of the patients correlated with the molecular findings and with the presumptive diagnosis of PVOD.

**Figure 4:**
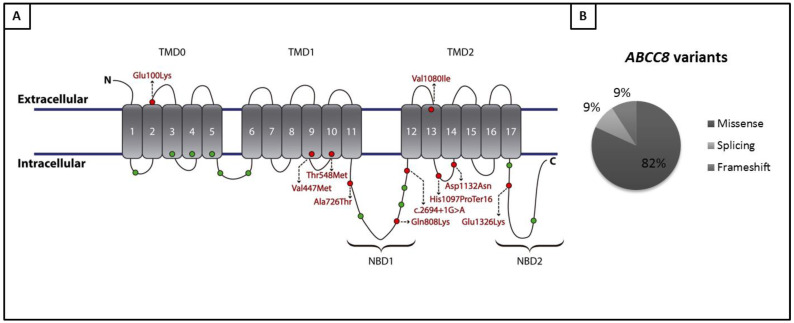
Variants detected in *ABCC8*. (**A**) Schematic representation of SUR1 (encoded by *ABCC8*) protein with its 17 transmembrane domains, and the variants detected in our cohort (red) and in Bohnen et al series (green). (**B**) Distribution of variants detected in *ABCC8* in our cohort based on the type of variant.

In *ABCC8*, a gene initially included with minimal evidence of relation with PAH, we found nine variants (Table 2, Figure 4), seven in IPAH individuals, one APAH, and one patient with CHD. All detected variants are distributed along SUR1, the protein encoded by *ABCC8*, so there are no hotspots in the gene, in concordance with previous reports [31] where the mechanism suggested was the loss of the ATP-sensitive potassium channel function. Functional assays by minigenes (data not shown), confirmed that the variant p.Asp1132Asn affects the splicing by exon skipping of exon 27 [32] and the variants p.(Glu100Lys), p.(Val477Met), and p.(Glu1326Lys) do not alter the splicing and maybe the pathogenic effect is not by splicing defects. In fact, variant p.(Glu1326Lys) has been found together with another variant in *NOTCH3* (NM_000435.2:c.6532C>T:p.(Pro2178Ser)), which means that further analysis must be performed to confirm the role of these changes.

Only one patient has been found to have a VUS in *CBLN2*, but further analysis needs to be done to elucidate the possible role of this gene in PAH.

In patients with PAH associated with CHD, we found ten variants in eight samples, three classified as pathogenic (two in *BMPR2* in two siblings and one in *TBX4*) and six VUS in *CPS1*, *ABCC8*, *SMAD5*, *SARS2*, *SMAD1*, and *NOTCH3* (Table 3). 

The two *BMPR2* mutation carriers came from unrelated families, inherited the variant from their healthy mother, and no additional candidate variants were detected in the NGS panel in both siblings, suggesting that there may be other factors influencing the PAH-phenotype in the siblings, such as second hits in other genes not included in the panel. One of these siblings is a male diagnosed with PH at 19 years of age. In the first work-up at diagnosis, an interventricular septal defect with a right to left shunt was observed. Right heart catheterization (RHC) confirmed suprasystemic PH. Considering these findings, ventricular septal defect closure was contraindicated, and PAH-targeted treatment was initiated. Clinical follow-up included a goal-oriented PAH therapy and the risk profile was assessed periodically according to the guidelines. Finally, he underwent bilateral lung transplantation four years after diagnosis.

His sister was also diagnosed with PAH at 26 years of age. At diagnosis, an interatrial septal defect with a left-to-right shunt was observed. In the RHC, PH with elevated pulmonary vascular resistance was confirmed. Dual oral PAH treatment was initiated, and she currently presents a low risk of death under oral therapy three years after diagnosis according to the hemodynamic parameters.

Previous studies suggested that pathogenic variants in *BMPR2* are present in up to 7.5% of patients with PAH associated with CHD [33,34]. Furthermore, Liu et al. observed that *BMPR2* pathogenic variants are more frequent in patients with pulmonary vascular disease (PVD) associated with CHD in comparison with those patients without PVD. To date, there is no effective method to predict the development of PH after the correction of a CHD. According to current guidelines, correction is contraindicated when PVD is established [35]. However, approximately 10% of patients with repaired atrial septal defect or ventricular septal defect develop PAH and therefore genetic background can contribute to explaining these differences. The role of germline mutations in these patients is unknown. These findings suggest that PAH mutations might contribute to the development of PAH in CHD patients. Genetic testing may allow us to identify high-risk patients. However, more studies are needed to assess the importance of genetic abnormalities in CHD.

In all patients where a pathogenic variant in *TBX4* was detected, the clinical expressivity was highly variable, including an initial suspicion of PVOD, interstitial lung disease, pulmonary vascular abnormalities, and CHD. In one individual, a variant in *SMAD1* has been also observed in addition to the *TBX4* variant. This variant in *SMAD1* was classified as VUS. He suffered with PAH associated with a non-repaired atrial septal defect. The variant observed in *TBX4* is a very infrequent variant with an extremely low frequency in the analyzed control populations (gnomAD exomes: 0.0000281, gnomAD genomes, 1000G, Kaviar, Beacon, ESP, and Bravo) although the majority of the in silico bioinformatic tools did not suggest a deleterious effect of the variant. Thus, the relationship of the variant detected in *SMAD1* and the phenotype of the patients remains unclear. The nonsense *TBX4* variant detected in this patient (*TBX4*(NM_018488.3):c.1018C>T:(p.Arg340*)) was reported last year by Galambos et al. [36]. In their study, the two siblings in whom the same variant was detected presented a clinically specific phenotype with transient patent ductus arteriosus, a patent foramen oval, and interstitial lung disease (ILD). Strikingly, one individual did not manifest pulmonary hypertension; reflecting the possible contribution of other genetic, epigenetic, or external factors that can contribute to the development of the disease. The presence of left-to-right shunt causes an overflow in pulmonary circulation that can lead to PH. However, this does not fully explain these complex cases. It is necessary to further investigate the possible involvement of *TBX4* in complex cardiac diseases such as CHD and ILD, and how it can contribute to increasing the risk of development of PH in these individuals.

Additionally, we found three variants in *CPS1*, one in a homozygous state, a gene for which neonatal susceptibility to PAH has been suggested with an increase of pulmonary arterial pressure after a surgical repair of congenital heart defects [37]. Furthermore, polymorphisms in *CPS1* have also been related to persistent pulmonary hypertension of the newborn (PPHN) [38]. All variants were classified as VUS and unfortunately, no parental samples were available for segregation analysis.

Between 15 and 30% of PAH cases are secondary to a CTD [39] such as systemic sclerosis or systemic lupus erythematosus. Five-year survival in PAH-CTD is around 44% in the US population [40]. In Spain, one-year survival in PAH-CTD is 81% [1,41]. There is increasing evidence of the contribution of genetic mutation in PAH-CTD, as previous reports suggested [17]. In our CTD-PAH cohort, we have found four variants (two classified as VUS in *ABCC8* and *NOTCH3*, and two as pathogenic in *TBX4* and *GDF2*) (Table 3), adding further evidence of the potential relation between variants in well-known genes for PAH and CTD-PAH. Furthermore, as far as we know, this is the first time a variant in *TBX4* is associated with CTD-PAH. All variants were found in different genes and de novo events were confirmed in segregation studies when available.

We have compared the clinical features of all patients with a confirmed genetic defect (Supplementary Tables S3–S5). We observed that those patients who carried a pathogenic or likely pathogenic variant had a higher mean pulmonary artery pressure (PAPm) and pulmonary vascular resistance (PVR), with a lower cardiac index. However, they were younger and less likely to respond to acute vasodilator testing. Regarding functional class, no statistical differences were observed in the clinical severity assessed by the WHO functional class. However, the six-minute walking test (6MWT) was statistically higher in the group of patients with pathogenic variants compared with the non-mutated group.

**Figure 5:**
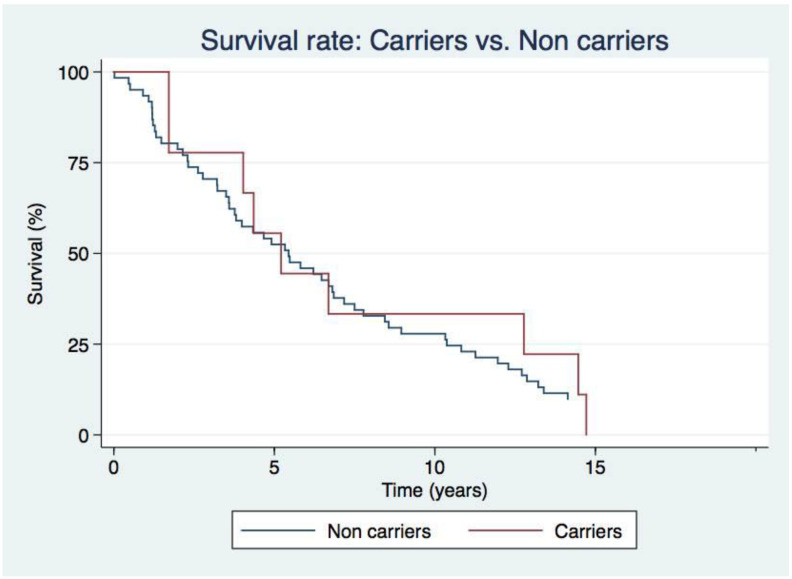
Survival analysis between carriers’ vs. non-carriers. Kaplan-Meier analysis did not reflect statistical differences between individuals with PAH and pathogenic or likely pathogenic variants and individuals without any pathogenic, likely pathogenic variant detected.

In terms of the overall survival rate, we have found no significant difference between both groups (Figure 5). Additional analysis of survival by Kaplan–Meier does not show any statistical differences between patients with different gene variants except for *EIF2AK4*, as we expected due to the severity of the associated PAH form. Also, we have not found statistical differences in survival between idiopathic, hereditary, and associated PAH forms, nor between carriers and non-carriers with APAH and PVOD (Supplementary Figure S2).

## 5. Conclusions

To summarize, we applied a custom personalized panel of genes for PAH diagnosis, which demonstrated to be very useful to confirm a presumptive diagnosis in cases with overlapping clinical features of PAH. The panel allows the identification of variants in the PAH related genes in a reasonable turnaround time for clinical purposes. We also detected variants in genes that have been recently described in PAH, and that could potentially be new therapeutic targets for personalized medicine. Lastly, in secondary PAH, we found variants that may influence the phenotype of the disease. Further studies to confirm the role of these variants are needed. 

## Figures and Tables

**Table 1:** Individuals in whom more than one candidate gene was detected. Variant prioritization showed that in five unrelated families more than one candidate gene was identified.

Patient ID	PAH Etiology	Variant 1	ACMG Classification	Variant 2	ACMG Classification
HTP070	IPAH	*BMPR2*:NM_001204.6:c.77-5_77-2delTTTA	P	*NOTCH3*:NM_000435.2:c.181C>T:p.(Arg61Trp)	VUS
HTP081	IPAH	*BMPR2*:Exon2 duplication	N/A	*NOTCH3*:NM_000435.2:c.1964A>G:p.(Asn655Ser)	VUS
HTP466	IPAH	*ABCC8:*NM_000352.4:c.3976G>A:p.(Glu1326Lys)	LP	*NOTCH3:*NM_000435.2:c.6532C>T:p.(Pro2178Ser)	VUS
HTP474	CHD	*ABCC8*:NM_000352.4(ABCC8):c.3394G>A:p.(Asp1132Asn)	VUS	*SARS2*:NM_017827.3:c.136G>A:p.(Glu46Lys)	VUS
HTP611	CHD	*TBX4*:NM_018488.3:c.1018C>T:p.(Arg340 *)	P	*SMAD1*:NM_005900.2:c.738G>C:p.(Met246Ile)	VUS

* Stop codon.

**Table 2:** Variants identified in *ABCC8.* ID and etiology of patients in which a variant in *ABCC8* was identified. Transcript for ABCC8 variant annotation was NM_000352.4.

Patient ID	PAH Etiology	Variant
HTP114	IPAH	*ABCC8*:NM_000352.4:c.1643C>T:p.(Thr548Met)
HTP88	IPAH	*ABCC8*:NM_000352:exon26:c.3288_3289del:p.(His1097Profs*16)
HTP151	IPAH	*ABCC8*:NM_000352.4:c.3238G>A:p.(Val1080Ile)
HTP159	IPAH	*ABCC8*:NM_000352.4:c.2422C>A:p.(Gln808Lys)
HTP162	IPAH	*ABCC8*:NM_000352.4:c.1429G>A:p.(Val477Met)
HTP466	IPAH	*ABCC8*:NM_000352.4:c.3976G>A:p.Glu1326Lys
HTP37	IPAH	*ABCC8*:NM_000352.3:c.579+5G>A
HTP78	CREST-PAH	*ABCC8:*NM_000352.3:c.2694+1G>A
HTP474	CHD	*ABCC8*:NM_000352.4:c.3394G>A:p.Asp1132Asn

**Table 3:** Variants detected in associated PAH forms (APAH). Description of variants found in patients with APAH, specifically with congenital heart disease (CHD) and connective tissue disease (CTD). ID: patient identifier, GT: genotype, ACMG: American College of Medical Genetics. Hom: homozygous; Het: heterozygous; P: pathogenic; VUS: variant of unknown significance.

ID	PAH Etiology	cDNA and Protein Position	GT	ACMG Classification
HTP501	CHD	*CPS1*:NM_001122633.2(CPS1):c.3047C>T:p.(Thr1016Met)	Hom	VUS
HTP536	CHD	*BMPR2*:NM_001204.6:c.2674delG: p.(Glu892Asnfs*4)	Het	P
HTP541	CHD	*BMPR2*:NM_001204.6:c.2674delG: p.(Glu892Asnfs*4)	Het	P
HTP474	CHD	*ABCC8*:NM_000352.4(ABCC8):c.3394G>A:p.(Asp1132Asn) *SARS2*:NM_017827.3:c.136G>A:p.(Glu46Lys)	Het Het	VUS
HTP558	CHD	*SMAD5*:NM_001001420.2:c.763A>G:p.(Ile255Val)	Het	VUS
HTP262	CHD	*NOTCH3*:NM_000435.3:c.6097C>G:p.(Pro2033Ala)	Het	VUS
HTP472	CHD	*CPS1*:NM_001122633.2:c.1036G>A:p.(Ala346Thr)	Het	VUS
HTP611	CHD	*TBX4*:NM_018488.3:c.1018C>T:p.(Arg340*) *SMAD1*:NM_005900.2:c.738G>C:p.(Met246Ile)	Het Het	P VUS
HTP551	CTD	*GDF2*:NM_016204:c.642G>A:p.(Trp214*)	Het	VUS
HTP355	CTD	*CPS1*:NM_001875.4:c.4252C>T:p.(Pro1418Ser)	Het	VUS
HTP452	CTD	*NOTCH3*:NM_000435:c.5203G>A:p.(Glu1735Lys)	Het	VUS
HTP564	CTD	*TBX4*:NM_018488.2:c.1112dupC:p.(Pro372Serfs*14)	Het	P
HTP78	CTD	*ABCC8:*NM_000352.3:c.2694+1G>A	Het	VUS

## Supplementary Materials

The following are available online at https://www.mdpi.com/2073-4425/11/10/1158/s1, Table S1: List of variants detected after prioritization. Table S2: List of variants detected after prioritization. Table S3: PAH Spanish Biobank cohort demographic and hemodynamic data. Table S4: PAH Spanish Biobank cohort demographic and hemodynamic data. Table S5: Clinical comparison between carriers’ vs. non carriers. Figure S1. Analyzed cohort of PAH. Figure S2: Survival analysis in PAH.
